# Towards a Secure Thermal-Energy Aware Routing Protocol in Wireless Body Area Network Based on Blockchain Technology

**DOI:** 10.3390/s20123604

**Published:** 2020-06-26

**Authors:** Zeinab Shahbazi, Yung-Cheol Byun

**Affiliations:** Department of Computer Engineering, Jeju National University, Jejusi 63243, Jeju Special Self-Governing, Korea; zeinab.sh@jejunu.ac.kr

**Keywords:** internet of things, blockchain, implanted sensors, smart contract, thermal/energy-aware routing protocol

## Abstract

The emergence of biomedical sensor devices, wireless communication, and innovation in other technologies for healthcare applications result in the evolution of a new area of research that is termed as Wireless Body Area Networks (WBANs). WBAN originates from Wireless Sensor Networks (WSNs), which are used for implementing many healthcare systems integrated with networks and wireless devices to ensure remote healthcare monitoring. WBAN is a network of wearable devices implanted in or on the human body. The main aim of WBAN is to collect the human vital signs/physiological data (like ECG, body temperature, EMG, glucose level, etc.) round-the-clock from patients that demand secure, optimal and efficient routing techniques. The efficient, secure, and reliable designing of routing protocol is a difficult task in WBAN due to its diverse characteristic and restraints, such as energy consumption and temperature-rise of implanted sensors. The two significant constraints, overheating of nodes and energy efficiency must be taken into account while designing a reliable blockchain-enabled WBAN routing protocol. The purpose of this study is to achieve stability and efficiency in the routing of WBAN through managing temperature and energy limitations. Moreover, the blockchain provides security, transparency, and lightweight solution for the interoperability of physiological data with other medical personnel in the healthcare ecosystem. In this research work, the blockchain-based Adaptive Thermal-/Energy-Aware Routing (ATEAR) protocol for WBAN is proposed. Temperature rise, energy consumption, and throughput are the evaluation metrics considered to analyze the performance of ATEAR for data transmission. In contrast, transaction throughput, latency, and resource utilization are used to investigate the outcome of the blockchain system. Hyperledger Caliper, a benchmarking tool, is used to evaluate the performance of the blockchain system in terms of CPU utilization, memory, and memory utilization. The results show that by preserving residual energy and avoiding overheated nodes as forwarders, high throughput is achieved with the ultimate increase of the network lifetime. Castalia, a simulation tool, is used to evaluate the performance of the proposed protocol, and its comparison is made with Multipath Ring Routing Protocol (MRRP), thermal-aware routing algorithm (TARA), and Shortest-Hop (SHR). Evaluation results illustrate that the proposed protocol performs significantly better in balancing of temperature (to avoid damaging heat effect on the body tissues) and energy consumption (to prevent the replacement of battery and to increase the embedded sensor node life) with efficient data transmission achieving a high throughput value.

## 1. Introduction

In the current era, health is the primary factor of concern for every person [1]. A large number of doctors are serving the patients day and night. Now, due to unhygienic food and polluted environment, diseases become very common [2]. Patients suffering from primary diseases such as diabetes, heart stock, high blood pressure, etc., require periodic monitoring for their health status, and this results in a splurge of money [3]. As the number of patients increase, a large number of doctors, hospitals, along with other medical personnel required for attending patients may also need to increase [3]. Moreover, the patients will require physical mobility, which could be difficult, and due to daily life activity, the patient is no more force to stay in a hospital. As the health factor is the primary concern for everyone; therefore, many healthcare organizations emphasize the use of Wireless Body Area Network (WBAN), e.g., World Health Organization (WHO) [4]. According to WHO, the main reason for deaths in the world by 2030 will be due to cardiovascular disease, heart diseases, and diabetes. The current survey describes the currently providing healthcare services to the patient with chronic diseases and patients with multiple health difficulties are rapidly increasing the cost of healthcare system [5]. Health care organizations make use of advanced information technology techniques and communication gadgets to overcome complex medical issues to reduce errors and minimize the overall cost, as per WHO providing healthcare services with advanced technologies for the exchange of sensitive information to overcome complex medical problems (e.g., data security and sharing etc.) to reduce error and for the continuity of healthcare providers [6]. It is, however, possible to utilize the latest technology for early identification and precaution of potential diseases that may take place later in the lives of people, e.g., telemedicine and e-health, etc.

Telemedicine is the use of advanced information technology and telecommunication that provide several healthcare services to patients [7]. Telemedicine enables remote treatment and helps to eliminate the distance barriers which improve medical access to distant rural communities. The current advancement in wireless communication and microchip technology brings the emergence of small, intelligent, wearable sensors able to use on the human body [8]. In WBAN, the implanted sensors required energy to communicate with each other; therefore, every sensor is equipped with a small battery. These sensors are utilized to measure the variation in patient vital signs and send data to the central node, which is responsible for coordinating nodes and send data to medical personnel for analyzing and real-time diagnosis. However, the security and privacy of medical data while sharing medical information is a challenging task in WBAN [9].

The current healthcare system does not meet the advanced systems requirements for an optimal operation because these systems have no reliable and consistent structure in terms of data sharing, security, and access control techniques. Hence, it is mandatory to design a secure wireless system to improve the data-access operation under the security and privacy of government regulations to assure accountability for monitoring and analyzing patient vital signs. Blockchain is a distributed ledger technology, which holds the secure log of translations [10]. The new technology blockchain revolutionized the existing healthcare platforms by adding its security, privacy, and data interoperability features. Blockchain technology replaces the current design approaches for data transmission between the user of the network and distributed ledger technology that support complete, immutable logs of transactions [11]. The blocks in blockchain consist of records that contain transaction details between the system and users. Blockchain technology can be beneficial in improving the telemedicine results in terms of security, transparency, audit, and trust.

The healthcare application integrated with implanted sensors for the acquisition of physiological data innovates the mechanism of traditional healthcare into WBAN [12]. WBAN is the branch of Wireless Sensor Network (WSN), which is used to integrate numerous implanted and wireless sensors to safeguard the remote health monitoring [13,14]. Therefore the designing of WBAN can add more value to human life in terms of timely detection and treatment [14]. The underlying aims of WBAN are to acquire physiological data from the patient(s) for steady monitoring that conclusively needs an efficient routing approach [13]. The reliable, secure, and efficient implementation of routing protocol is a challenging task in WBAN due to its unique characteristics and limitations, such as energy reduction or overheating of implanted sensors nodes. The heat-rise and energy depletion influence the constancy of a network, hence the data is transmitted through various paths in WBAN.

During the last decade, several studies have been carried out, and many system have been developed on WBAN. The various characteristics of WBAN have raised the number of issues in a different layer of WBAN. At physical layer problems of interoperability, temperature control, changing topology, interference, fault acceptance, security, etc. The issues related to the MAC layer are dynamic channel assignment, control packets overhead, protocol overhead, throughput, synchronization, delay control, etc. The problems related to the network layer are mobility, localization, traffic control, temperature and heat control, optimum routing, etc. [15].

The main contribution of the paper is as follows:

- To design an optimized (minimum heat-rise and maximize energy conservation to enhance network lifetime) routing protocol for WBAN by considering primary metrics, i.e., node temperature, residual energy for next-hop selection.
- To build a self-adaptive routing algorithm, capable of selecting alternative forwarder nodes in case of unavailability of a node due to heat and residual energy.
- To develop a scalable system which fulfills the requirements of practical healthcare application, integrated with several IoT healthcare based on blockchain.
- Lightweight: The developed system provide the cross-platform communication between the WBAN and blockchain network using RESTful API.
- Transparency: The proposed blockchain-enabled WBAN system maintains the integrity of the patient’s vital signs data, and also provides unauthorized access to the personal medical information.
- Scalability: The proposed system fulfills the requirements of implant in-body or on-body several implanted sensors to diverse constrained networks to a single blockchain.
- High Throughput: The design platform is based on permissioned blockchain network, where all the set of entities are authorized and authentic. Hence, consensus protocol such as crash fault tolerant (CFT), and byzantine fault-tolerant (BFT) can be used to elevate the throughput of the network.

In this article, we propose a blockchain-enabled WBAN based self-adaptive thermal-energy aware routing protocol. Our proposed system is based on blockchain where miniature sensors are implanted in/on the patient body in such a way that these healthcare senors get the physiological data and share with the certified user of the system within the blockchain network. Furthermore, the design system also contributes with two main constraints, i.e., implanted devices in the patient body, which can damage adjacent body tissues as a continual transfer of physiological data and node replacement due to energy conservation. Therefore to establish optimal (minimum heat-rise and maximize energy conservation to enhance network lifetime) routing protocol for WBAN by considering primary metrics, i.e., node temperature, residual energy for next-hop selection. Furthermore, the self-adaptive routing algorithm is capable of selecting alternative forwarder nodes in case of the unavailability of a node due to heat and residual energy. The results show that by preserving residual energy and avoiding overheated nodes as forwarders, high throughput is achieved with the ultimate increase of the network lifetime. Moreover, the composer rest-server is used to create the RESTful API to trigger the application-specific services provided by the blockchain network. The access control rules offered by the smart contract are used to authenticate and authorized the users and information related to patients and other medical data, e.g., only the authorized medical personnel are allowed to manipulate and access the patient medical information. The traditional database is replaced by the CouchDB to overcome the issue of redundancy and support large transactions of data. Therefore, each peer in the network is equipped with CouchDB to support file storage within the entire blockchain network. Castalia [16], a simulation tool, is used to evaluate the performance of the proposed protocol, and its comparison is made with Multipath Ring Routing Protocol (MRRP), thermal-aware routing algorithm (TARA), and Shortest-Hop (SHR). Likewise, Hyperledger Caliper [17] is used to evaluate the performance of the blockchain system in terms of transaction throughput and latency. Evaluation results illustrate that the proposed protocol performs significantly better in balancing of temperature (to avoid damaging heat effect of the body tissues) and energy consumption (to prevent the replacement of battery and to increase the embedded sensor node life).

The remaining of this paper is categorized as follows: Section 2 gives the brief on WBAN approaches and gives the details insight on related projects and their limitations. Section 3 explains the thermal/energy-aware routing protocol based on blockchain for secure monitoring of patient vital sign. Section 4 presents the simulation and performance evaluation of the proposed blockchain-enabled routing protocol. Section 5 concludes the paper with future research directions.

## 2. Literature Review

In WBAN, routing is a challenging task because of the limited available resources like limited memory, computational power, and energy source [18]. Energy consumption is greatly affected by the Radio Frequency (RF) at the physical layer of sensor nodes in WBAN. Besides, the residual energy of sensor nodes can be increased by the MAC protocols by controlling the duty cycle of the RF part. Regardless of this fact that physical and MAC layer protocols improve network communication, still, some other requirements, including end-to-end packet delivery, addressing techniques, and route determination methods, are not discussed. Here, the network layer is the optimal option to address these issues, and consideration of network layer routing protocols is very significant in terms of energy conservation [19]. WBAN is a subset of WSN; however, the characteristics and specifications of WBAN are different from WSN. Therefore, the designing of an efficient WBAN routing protocol needs to be considered carefully than WSN. Various important factors are required to be considered for the WBAN routing protocols, i.e., heterogeneous network nature (due to specific physiological data), energy consumption (due to higher transmission rate), temperature-rise (due to a high number of transmissions), coverage area (due to low transmission dimension), mobility (due to postural movements) and quality of service (due to priority-based data) [20]. Among all stated characteristics, the most crucial factor is related to the temperature-rise of implanted sensors that adversely affects the performance of WBAN. The temperature of a sensor node rises due to radiation absorbed and energy consumption by the antenna that causes the damage to the heat-sensitive areas of the body [21] and can affect body tissue surrounding the sensor nodes [22]. Therefore, careful research is needed to design a routing algorithm for WBAN to prevent damage of body tissue due to the temperature-rise of a node. The second important consideration for WBAN routing is to deal with energy consumption issues as the replacement of sensor nodes (particularly implanted sensor nodes) is not feasible. Energy consumed during sensing and monitoring of data is less than the energy used during communication between the devices [23]. Moreover, a limited communication range for data transmission prevents the energy consumption, and the proposed routing algorithm is required to be capable of choosing the optimal path among the multiple available paths. Hence, intelligent routing (considering temperature-rise and residual energy) can enhance the life-span of a network.

### 2.1. Classification of WBAN Routing Protocol

WBAN can be divided into six categories, i.e., cross layered routing protocol, quality of service aware routing protocol (QoS), cluster-based routing protocol, temperature-aware routing approaches, postural movement-based routing protocol and energy-aware routing routing protocol.

#### 2.1.1. QoS Routing

The QoS routing protocols work based on QoS metrics. The QoS routing is a modular-based protocol where synchronization and network density make it difficult to enhance the QoS protocol. In WBAN, patients’ data (critical or normal) is considered very important and transmitted efficiently and securely based on QoS metrics. The QoS routing protocols can be divided into two sub-categories, such as delay-tolerant and reliability-based protocol, where the reliability-based protocol enhances the throughput and minimize the delay and delay-tolerant protocol assure the packets delivery in time. The following are the few QoS-based routing protocols presented such as RL-QRP protocol [24], ENSA-BAN [25], multi-hop protocol using cost function [26], CDR [19], ARBA [27], QPRR [23], and QPRD [28].

#### 2.1.2. Postural Movement Routing

Communication in WBAN is affected by the postural movement of the human body. The communication between nodes is usually disconnected by frequent body movement. Several research studies have been made in order to define the cost function for optimal routing to sink. Some of the postural movement-based routing protocols are ETPA [29], PSR [30], DVRPLC [31], and PRPLC [18].

#### 2.1.3. Energy-Aware Routing

Maximizing energy conservation of nodes is a challenge in WBAN. The size of the battery in WBAN is compact and small with little energy consumption. Several energy-aware routing protocols have been proposed in order to preserve the power for the extended life of wireless networks. Many of the existing power-saving approaches are used to evade message headers, decreasing the frequency of sending network control messages and redundant retransmissions. A few of energy-aware routing protocols are Co-CEStat [20], MEPF [21], RSSI [22], DARE [32], ESR [33], and SIMPLE [34]. Table 1, summarized the pros and cons of the existing temperature-aware routing protocol.

#### 2.1.4. Temperature-Aware Routing

In body sensor network designing, interference and absorption of antenna radiation are the crucial tasks. In WBAN, the human body can be affected by the radiation emitted for the sensors implant in or on the human body [35]. In [36], authors presented a model for the effect caused by implanted sensors on the human body during communication. On the basis of observation, authors have proposed a rate controlled recommendation model for WBAN designing. On the other hand, contrary to rate control, during the designing of Intra-WBAN, thermal-aware routing techniques are very important to consider. Various thermal-aware routing protocols have been proposed to avoid the temperature-rise and radiation that cause damaging effects to the human body. The existing thermal-aware routing protocols presented in the literature are $M^{2}E^{2}$ [37], RE-ATTEMPT [38], (TMQoS) [39], M-ATTEMPT [38], ATTEMPT [38], HPR [40], THSR [41], RAIN [42], LTRT [43], LTR [44], ALTR [44], and TARA [45]. Table 2, summarized the pros and cons of the existing temperature-aware routing protocol. Table 2, summarized the pros and cons of the existing temperature-aware routing protocol.

#### 2.1.5. Cluster Routing

In WBAN, temperature and energy are the main factors to be evaluated. Cluster-based routing is the type of WBAN routing which aims to decrease the energy consumption and maximize the life-cycle of a network. In cluster-based routing, the node energy consumption is minimized by breaking the networks into portions, and each portion represents a cluster. These clusters are used for a communication head using a cluster head (CH). The CH is responsible for forwarding, aggregating, and collecting the data. The existing cluster routing protocols discussed in the literature include hybrid indirect transmission (HIT) [46], AnyBody [47], Cluster-based body are protocol (CBBAP) [48].

#### 2.1.6. Cross Layered Routing

The cross-layer routing protocol is mainly used for the development of wireless sensors networks. The fundamental responsibility of the cross-layer approach is to provide synchronization among the layer without affecting the MAC layer functionality. Moreover, cross-layer routing protocols significantly enhance the network performance and resolve issues related to network and MAC layer. Cross-layer routing protocols are divided into four categories, i.e., Biocomm and Biocomm-D [49], TICOSS [50], CICADA [51], and WASP [52].

#### 2.1.7. WBAN-Enabled Blockchain Approaches

Griggs et al. [53] presented an ethereum based protocol where a smart contract is used to establish communication between sensors and intelligent devices and store data in the blockchain. The real-time monitoring support provided by the smart contract enables medical personnel to get up to date medical records of the patient. Khalid et al. [54] describe the approach to integrate the blockchain with WBAN using multiple IoT devices. The blockchain provides trust and reliability between the parties while transforming data. Furthermore, the presented system also discussed several challenges that can be solved by concatenating both technologies like blockchain and WBAN. Rani et al. [55] introduced a technique to incorporate WBAN with blockchain for securing electronic health records. The sharing of medical data between the parties is made secure and reliable using blockchain. Moreover, the blockchain provides authentication and authorization rules to validate each transaction within the network. The data is gathered using ECG and movement sensor plants on the human body. The QoS support is also enabled for remote monitoring, interoperability, and correspondence. Gervais et al. [56] presented an approach for securing the blockchain and WBAN integration. The proposed certificates authenticated key agreement (CLAKA) approach not only used to overcome the problem of a single node of failure in the network but also to prove authentication and authorization with common sharing of key between blockchain nodes and WBAN user. Similarly, Junchao et al. [57] presented an e-healthcare system based on blockchain integrated with WBAN, which provides security for patient data. The proposed system consumes fewer hardware resources while maximizing the data protection level. The remote monitoring based on the blockchain offers the data interoperability, where data are shared among different users of the system like pharmacy personnel, medical center, health insurance, and emergency service.

In WBAN, sensor nodes of a minimal size are usually implanted in or on the human body. The heat-rise of implanted sensors nodes and the energy consumption are the two significant challenges required to be dealt through efficient routing. As aforementioned, these WBAN enabled blockchain approaches did not consider the temperature-rise and energy-consumption for next-hop selection, which ultimately damages adjacent body tissues in case of continual transfer of physiological data. Moreover, none of the existing approaches minimized energy conservation to avoid frequent replacement of implanted nodes in WBAN. Additionally, many of the strategies discussed in the literature review are related to the management of health records or data sharing among different healthcare providers. Regardless, not any of the thermal/energy-aware approaches will address minimum heat-rise and maximize energy conservation in WBAN using hyperledger fabric. To the best knowledge of the author, there has been no functional routing protocol that is used to optimized heat-rise and energy conservation in WBAN based on blockchain to enhance network life-span built so far.

## 3. System Model

**Figure 1:**
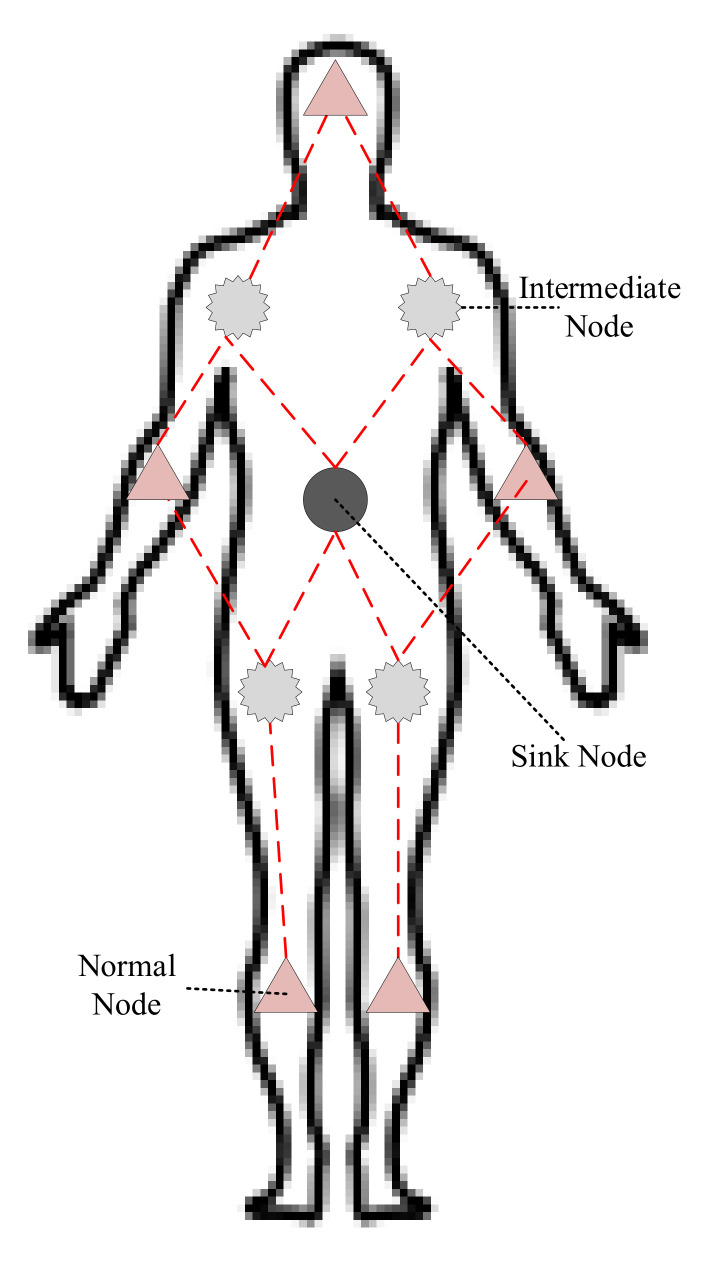
Deployments of nodes and their communication.

Reliable and stable data transmission with low energy consumption and the least temperature-rise of each sensor node is significant in WBAN. One of the primary goals of this research is to intelligently operate single-hop and multihop transmission to enhance the network efficiency in domains of energy and temperature. In our proposed model, the sink is planted in the middle of the human body, as shown in Figure 1.

WBAN is a heterogeneous network, the deployment of nodes in/on the body is application dependent; therefore, in our scheme, we are taking 9 or 12 implanted sensor nodes. The nodes at a distance of one-hop from the sink can communicate directly with the sink while distant nodes carry out their communication using intermediate nodes (multihop). We have taken some assumptions in our proposed routing protocol listed below:

- Every node in the network are fixed and implanted
- The sink node’s responsibility is to acquired data from all the neighbouring sensor nodes. The software and hardware resources are sufficient with a steady energy supply.
- The transmission range and power are static for every sensor.
- The sink node is the final destination of each sensor node.
- Each sensor node is generated by the fixed size of the packet.
- The multihop path is used to forward the data to the destination.
- Optimum path selection is made by considering two constraints of WBAN, i.e., temperature- rise and energy consumption of a node from source to destination.

Energy consumption and temperature-rise issues are addressed in our approach by selecting the forwarding node with the minimum temperature and maximum energy. Moreover, the node having a temperature more than the threshold value of both energy and temperature is also avoided.

### 3.1. Proposed WBAN Blockchain Platform System Architecture

Blockchain is a fundamentally transparent platform where the interaction between users and smart contracts modeled as crypto-signed but unencrypted transactions is visible to all participants in the blockchain network. The core functionality of blockchain technology is to provide solutions for the sharing of sensitive data. Data is only accessible to a limited number of recipients. The blockchain data is only unlocked due to the cryptographic artifacts. The privacy of the data is important, so due to the blockchain, this can be achieved when designing a new platform with an additional layer of encryption for the enhancement of the patient’s vital signs data. We developed a customized solution that enables user- and group-based secret sharing by utilizing access control rules based on hospital privacy and security requirements so that data can be shared across the user of the system in the healthcare ecosystem.

**Figure 2:**
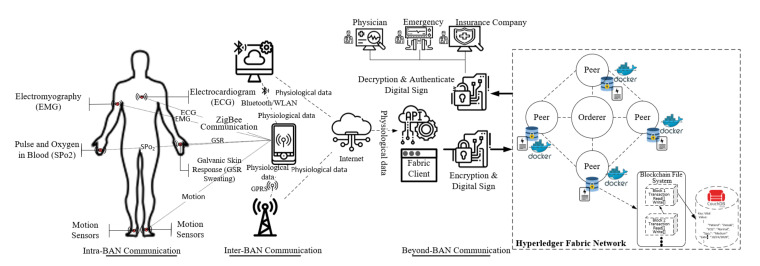
Blockchain-enabled WBAN architecture.

The proposed blockchain-enabled ATEAR routing protocol is comprised of two modules, i.e., the ATEAR routing protocol and blockchain. Figure 2 shows the overall architecture of the design system. The proposed method is divided into three tiers, i.e., intra-BAN, inter-BAN, and beyond-BAN communication. The process starts from intra-BAN communication in which all sensor nodes implanted in or on the human body for collecting physiological data and send it to (personal server) PS within a two-meter transmission range. The data then passed through in tier-2 through wireless technology such as Bluetooth, WiFi, and ZigBee. As energy is the dominant factor in sensors and PD, so these sensors and PD can be directly connected to access point, wired, wireless, and as per the application requirement. Similarly, in inter-BAN communication, PD is connected with one or more access points. The primary purpose of Tier-2 is to interconnect sensors with the various network to utilize their functionality. The inter-BAN communication paradigm is divided into two categories, infrastructure-based architecture ad-hoc based architecture. In tier-3, we add the blockchain model to provide data authentication, privacy, and security. For data authorization and authentication, we defined access control rules (ACL) for the user of the system. These rules depict and regulate which roles/users are allowed to perform read, delete, update, and create in the blockchain business network model. Furthermore, the designed smart contract also contains transaction processor function, which is implemented for various services like update patient information, share the record with doctors, physicians, and insurance companies. Similarly, by using the composer-rest-server, we develop the Restful API to expose the blockchain services to the client-end. The proposed system provides data interoperability between various stakeholders in the healthcare ecosystem.

### 3.2. Adaptive Thermal-Energy-Aware Routing Protocol in WBAN

The perseverance of energy and evasion of temperature-rise are the primary concerns for the WBAN network life. To achieve efficiency and stability of the network, these two challenges need to be dealt with collectively. The potential problems are related to send data packet (from source to destination) with less energy consumption and less temperature for every communication. The existing routing approaches for WBAN addressed these issues individually. However, frequent communication using an optimal route with high energy and low temperature will ultimately result in energy exhaustion and overheating of that route or death of a node. In this research, this problem is resolved by presenting a protocol “ATEAR—Adaptive Thermal-/Energy-Aware Routing in WBAN”. This protocol provides the mechanism of selection of optimal multihop paths among multiple available routes to avoid frequent partitioning of WBAN. This optimization is achieved by discovering multiple relay nodes from the source to destination, and each node is given a cost value based on the temperature and energy metric. Every time before sending the data, the forwarding node is selected depending upon the cost function. Thus, communication will be carried out using multiple options of the nodes avoiding a single route or node used continuously. ATEAR is a reactive source protocol initiated by the source node to forward the data using alternative options. Our proposed ATEAR scheme constitutes two phases, i.e., the initialization phase and data transmission phase.

#### 3.2.1. Initialization Phase

The initialization process is initiated by the destination node to obtain the distance of each node from destination in terms of the number of hops. This phase involves the organization of the network into levels. At the end of this phase, each node is assigned a ring level value; this value of the level indicates its distance from the sink. At the beginning of this phase, a packet is broadcast by the sink with a ring level value of 0 in the packet, and this packet is received by all of its neighbours. The nodes that receive this packet will update the ring level field by incrementing 1 in the ring level value and rebroadcast the packet. This operation of rebroadcasting and updating of the ring level value continues until the end of this phase until each node acquires its ring level. The format of the setup packet in initialization phase includes sourceID, destinationID, temperature, ringlevel, and energy. The sourceID indicates the unique ID of the node which broadcasts the setup packet. Destination ID represents the destination address, energy and temperature field indicates the energy and temperature of the sender node.

**Figure 3:**
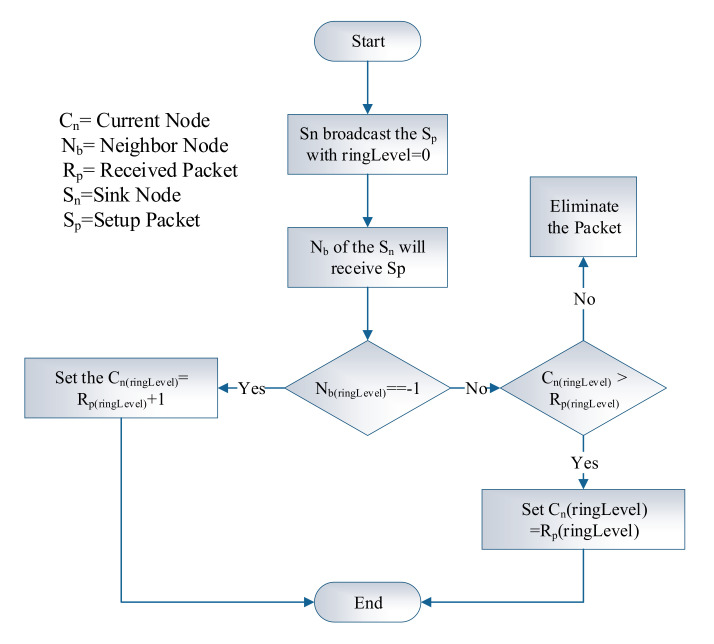
Initialization phase.

In the initialization phase, the primary goal is to generate the ring levels for all nodes—every node broadcast this setup packet to explore its neighbours. On receiving the setup packet, a sensor node considers itself in level n if the hop count is n from the sink. If a node receives a packet with a smaller value of ring level, then it will update according to the new ring level. The algorithm of the initialization phase is given below, and the flow chart is shown in Figure 3.

This phase is responsible for acquiring available sensors nodes distance from sink with the current statistics of temperature and energy of every node. The appropriate route selection is based on the stability of route, which depends on the node energy and temperature. The multiple forwarding nodes are available, the cost is evaluated, and selection of node depends upon this calculated cost value. The algorithm of the initialization phase is given below, and the flow chart is shown in Algorithm 1.

**Algorithm 1:** Initialization Phase

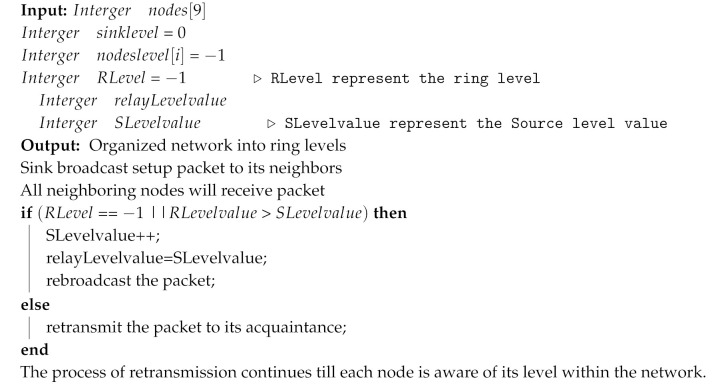



#### 3.2.2. Data Transmission Phase

In this proposed protocol, the data is forwarded using relay nodes rather than of having direct communication between the source node and destination node to reduce energy consumption and temperature rise. The source node broadcasts the data packet with its ring level. On receiving the packet, the ring level of the relay node is compared with the received data packet ring level. For instance, if the value of the relay node ring level is less and the ID of the relay node, then the packet is processed and broadcast; otherwise, the data packet will be discarded. This operation will continue until the packet is destined to the sink node. If the ring level value of the received data packet is less than the neighbour node ring level value, then the energy, temperature, and IP address are stored by the neighbour node and discard the packet. Afterwards, the information is used to select the optimal next hop for the forwarding data packet.

The neighboring nodes only received data packets form the current node whose path in terms of distance to destination is more than itself (current node). Hence, only relay nodes (Pr) among the neighboring nodes accept the setup packet which fulfills Equation (1).

(1) $$d\left(N_{src},N_{dest}\right)<d\left(P_{r},N_{dest}\right)$$

where $d(N_{src},Pr)$ is the distance between the sending node and the relay node.

Every node in the network is categorized based on energy and temperature. These selected nodes later act as a next hop, which is used to forward the data packet. The least rank value node has the highest chance to get selected as a next hop for the source node as presented in Table 3. All of the nodes within the network are ranked on the basis of their temperature and energy. These nodes in the network are categorized based on energy and temperature. Afterwards, the categorized nodes are used as next-hop to forward the data packet. The priority is given to the least categorized node in terms of ranking as a next-hop selection for the source node.

**Figure 4:**
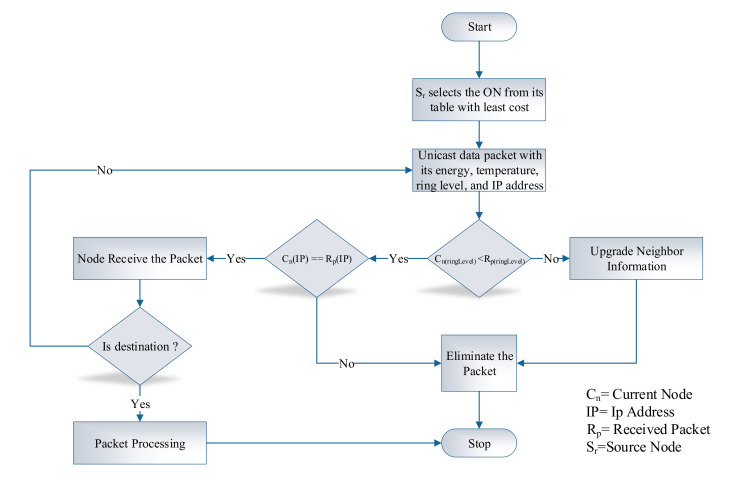
Initialization phase.

The rank value assessed for the next hop selection which is based on the following Equations (2)–(5).

(2) $$R_{TN}=min\left(N_{T}\right)forallnodes$$

(3) $$R_{EN}=max\left(N_{E}\right)forallnodes$$

(4) $$R_{AN}=R_{TN}+R_{EN}forallnodes$$

(5) $$O_{N}=min\left(R_{AN}\right)$$

where, $R_{TN}$ and $R_{EN}$ represent the rank given to the temperature and energy of each node, respectively. However, the node selection criteria is based on rank value denotes as RAN. Finally, $O_{N}$ is the optimal node that holds the node with the minimum rank value. The data packet format in this phase includes sourceID, destinationID, temperature, energy, ringLevel, selectedID, and data. The sourceID represents the node which broadcast the data packet, whereas destinationID is denoted as destination address, and temperature represents the temperature of the sender node. Similarly, the energy field indicates the energy of the sender node, and the selected IP address is the address of the relay node. The flow chart of the data transmission phase is illustrated in Figure 4, whereas the algorithm for the data transmission phase is presented in Algorithm 2.

**Algorithm 2:** Data transmission phase

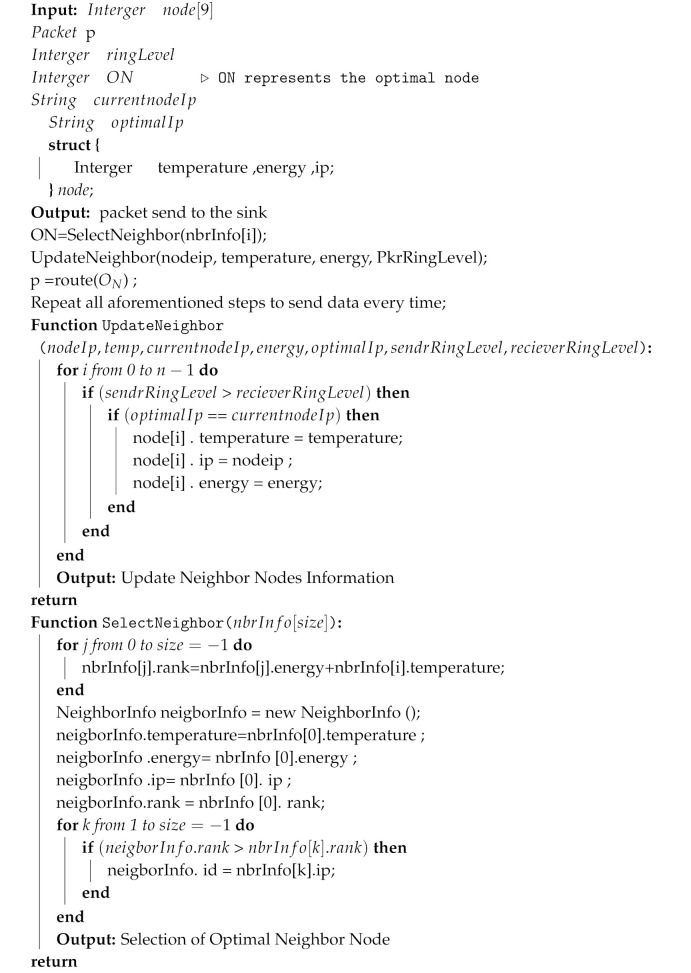



## 4. Simulation and Performance Evaluation

In this section, we have discussed the simulation setup and the obtained results of the proposed blockchain-enabled WBAN routing protocol. For simulation, we have used OMNeT++, a simulator tool, for the performance evaluation and comparison with other related routing approaches. OMNeT++ [58], the simulator tool, is selected due to its significant features, including open-source, GUI library, academic version, easy debugging, portable, and various application-specific frameworks.

### 4.1. Simulation Set-Up/Network Model

In this study, two different scenarios by changing the number of nodes were considered to analyze the performance of the WBAN network. We have considered 9 or 12 sensor nodes that are implanted in the human body. A static and rechargeable sink was placed at the center of the body, whose responsibility is to collect and forward all sensed data from all the sensor nodes to the external server or PDA. However, communication afar from the sink was not considered for this protocol. We assumed all the sensor nodes were homogeneous in terms of their computational power, sensing ability, initial energy level, and the temperature. The packet size considered in this work was of 296 bits. In order to analyze the performance, the proposed protocol was compared with the existing Multipath Ring Routing protocol. In our proposed protocol, the selection of the relay node was premeditated and is based on residual energy and temperature of the node. The simulation setup along with the energy values and temperature values are given in Table 4

### 4.2. Performance Evaluation Parameters

Evaluation metrics used for the proposed work are given here:

- **Average Throughput**: Throughput is the rate at which information was sent through the network.
- **Temperature rise**: Represents the increase in the temperature of each node during data transmission.
- **Energy Consumption**: Energy consumed during the simulation time.
- **Simulation time**: The time period during which a simulation ran over the simulator
- **Average Temperature rise**: Represents the average increase of temperature for the overall network.
- **Average Energy Consumption**: Average energy consumption indicates the average decrease or consumed during the overall network during simulation time.

### 4.3. Simulations Results

#### 4.3.1. Average Temperature Rise versus Simulation Time

**Figure 5:**
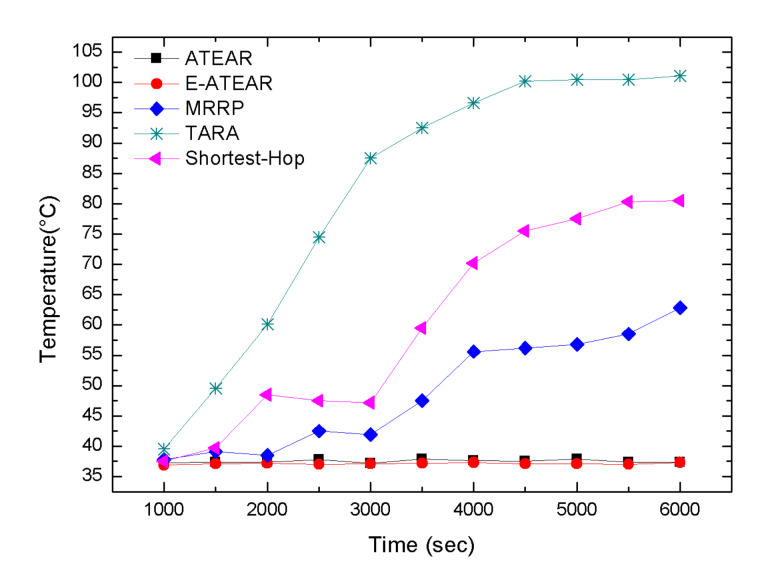
Average temperature rise versus simulation.

Figure 5 shows the behavior of a network in terms of the average temperature for five protocols, i.e., Multipath Ring Routing Protocol (MRRP), the proposed protocol ATEAR, Enhanced ATEAR protocol, thermal-aware routing algorithm (TARA), and Shortest-Hop (SHR). At the beginning of the simulation, each node within the network has the same temperature, i.e., 37 ${}^{{}^{\circ}}$C. The graph in Figure 5 shows that the simulation started from 1000 s and ended at 6000 s. The result demonstrates the thermal-aware feature of the ATEAR and Enhanced ATEAR protocol. The average temperature of the network with ATEAR reached 37.02 ${}^{{}^{\circ}}$C at 1000 s and 37.749 ${}^{{}^{\circ}}$C at 6000 s. Contrary to this, with MRRP, TARA, and SHR initially the average temperature rose to 37.8 ${}^{{}^{\circ}}$C, 38.8 ${}^{{}^{\circ}}$C, and 39.8 ${}^{{}^{\circ}}$C at 1000 and in the end at 6000 s it was 62.8 ${}^{{}^{\circ}}$C, 80.8 ${}^{{}^{\circ}}$C, and 101.8 ${}^{{}^{\circ}}$C (not feasible for implanted nodes). ATEAR significantly performed better in terms of the overheating of the sensor nodes. Furthermore, the average temperature of Enhanced ATEAR was also investigated, which rose to 37.25 ${}^{{}^{\circ}}$C at 1000 s and 37.70 ${}^{{}^{\circ}}$C till the end of simulation at 6000 s. Thus, Enhanced ATEAR performed slightly better than ATEAR and much better than MRRP. Consequently, the temperature peaked in the above graph showing the unawareness of MRRP, TARA, and SHR towards the overheating of the embedded sensors while ATEAR and Enhanced ATEAR sensibly avoided it. The low peak of the temperature of ATEAR and E-ATEAR is due to the selection of the forwarder node with the minimum temperature-rise.

#### 4.3.2. Average Energy Consumption versus Simulation Time

**Figure 6:**
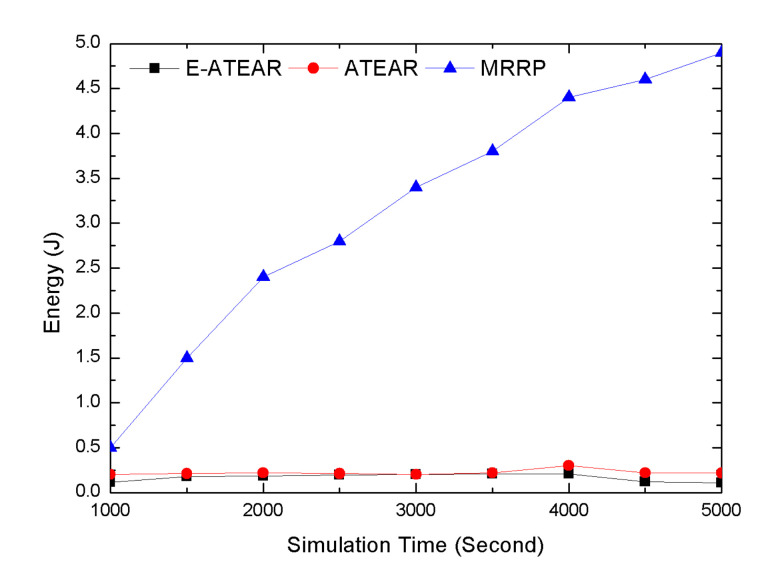
Average energy versus simulation time.

Average energy consumption over different simulation times is shown in Figure 6. The comparison of three protocols, i.e., MRRP, ATEAR, and Enhanced ATEAR, indicates average network energy consumption at different simulation times. The initial energy of each node was 0.3 j. From results, it was observed that ATEAR consumed 0.139 j, MRRP consumed 0.184 j, and Enhanced ATEAR (E-ATEAR) consumed 0.125 j of energy at 1000 s. At the end of simulation at 5000 s, the energy consumption of ATEAR, MRRP, E- ATEAR protocols reached 0.184, 2.456 j (which was practically not possible for implanted sensors), and 0.184 j respectively. This investigation showed the performances of ATEAR and Enhanced ATEAR were far better than MRRP. The neglected factor of energy consumption in MRRP was overcome in ATEAR and Enhanced ATEAR.

#### 4.3.3. Temperature Variation versus Simulation Time

**Figure 7:**
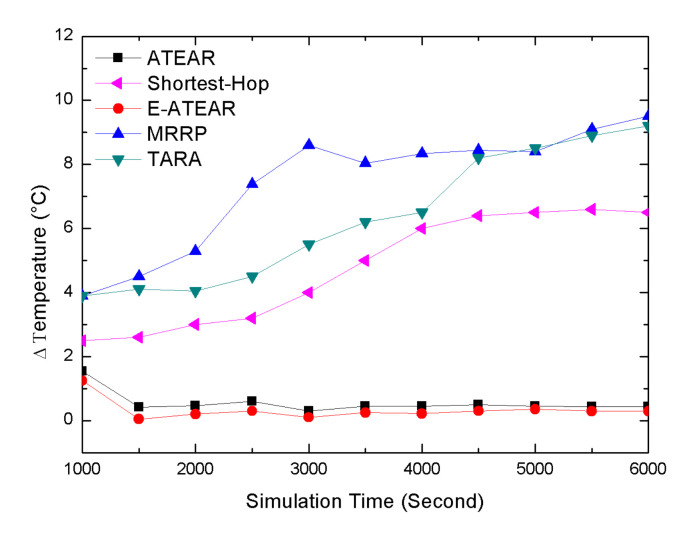
Temperature variation versus simulation time.

Temperature variation indicates the change in temperature (Δ temperature) rise over different simulation times. The graph is given in Figure 7 represents Δ temperature as the simulation time increases. Initially, the value of Δ temperature for ATEAR, MRRP, SHR, TARA and Enhanced ATEAR increased by 1.54 ${}^{{}^{\circ}}$C, 3.9 ${}^{{}^{\circ}}$C, 2.4 ${}^{{}^{\circ}}$C, 3.97 ${}^{{}^{\circ}}$C and 1.25 ${}^{{}^{\circ}}$C respectively. It can be clearly noticed that because of balancing of temperature among the nodes, the change of temperature in ATEAR and Enhanced ATEAR was less than the MRRP, SHR, and TARA. Similarly, as the simulation time increased to 6000 s, the change in temperature observed in ATEAR, MRRP, SHR, TARA, and Enhanced ATEAR was 0.43 ${}^{{}^{\circ}}$C, 6.98 ${}^{{}^{\circ}}$C, 10.5 ${}^{{}^{\circ}}$C, 8.05 ${}^{{}^{\circ}}$C and 0.29 ${}^{{}^{\circ}}$C. The Δ temperature for ATEAR was 0.43 ${}^{{}^{\circ}}$C, which means the average temperature of the network slightly changed after reaching the threshold value; Δ temperature for Enhanced ATEAR was 0.001 ${}^{{}^{\circ}}$C because the temperature of a node decreases after some time when no data forwarding was taking place. The Δ temperature in MRRP, SHR, TARA held a higher value because of thermal unawareness.

#### 4.3.4. Energy Variation versus Simulation Time

**Figure 8:**
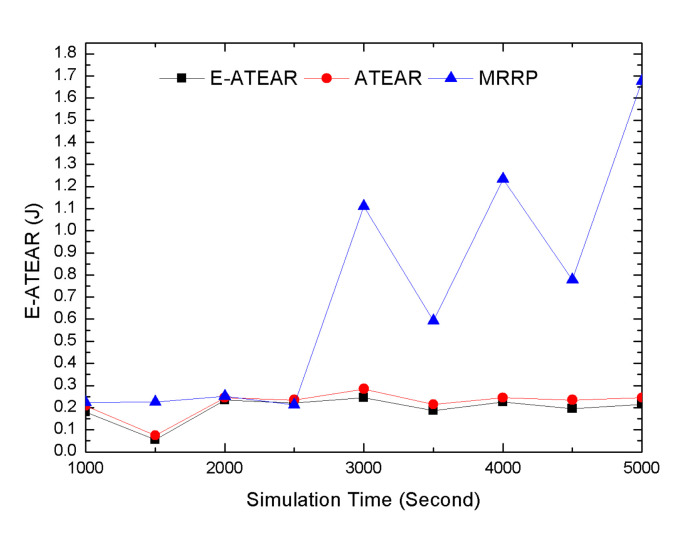
Energy Variation versus simulation time.

Energy variation represented by Δ Energy is given in Figure 8. In the first simulation at 1000 s, the Δ energy for ATEAR, MRRP, and Enhanced ATEAR was 0.161 j, 0.023 j, and 0.175 j, respectively. Similarly, at 5000 s, this change in energy was 0.443 j, 1.677 j, and 0.456 j for ATEAR, Enhanced ATEAR. With the increase in simulation time, more energy consumption occurred, so the value of change in energy was also increased, but it can be seen that ATEAR balanced the energy consumption showing less Δ energy value. Enhanced ATEAR harvest the energy, so it had the best result among these three protocols.

#### 4.3.5. Average Temperature Rise Consumption with Different Node Density

**Figure 9:**
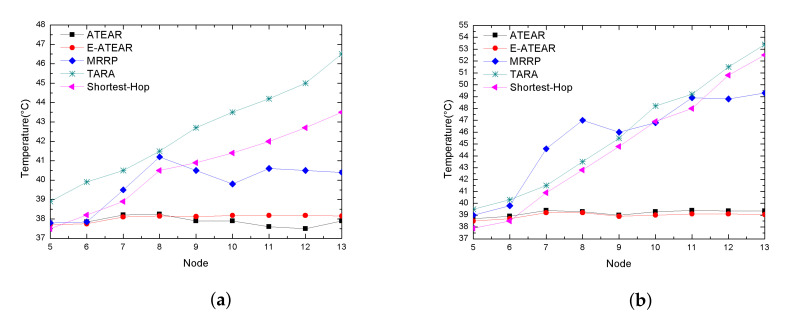
Average temperature rise consumption with different node density. (**a**) Simulation time = 300 s; (**b**) simulation time = 1000 s.

Figure 9 represents the comparison of temperature-rise of ATEAR, Enhanced-ATEAR, MRRP, SHR, and TARA protocols within networks having a different number of nodes, i.e., 5–13 at simulation time 300 s in Figure 9a and 1000 s Figure 9b respectively. It can be observed from both graphs that even by changing the size of the network performance of ATEAR and Enhanced-ATEAR was better than MRRP, SHR, and TARA in terms of average temperature rise. At 300 s simulation time in Figure 9a, initially, the temperature of the network rose in case of MRRP, SHR, and TARA is 37.8 ${}^{{}^{\circ}}$C, 38.3 ${}^{{}^{\circ}}$C, and 37.5 ${}^{{}^{\circ}}$C even at 300 s and as the number of nodes increased, the temperature reached 40.336 ${}^{{}^{\circ}}$C in the cases of MRRP, SHR, and TARA. Contrary to this, it can be seen that both ATEAR and Enhanced-ATEAR protocols showed consistent temperature rise both at 300 s and 1000 s with changing node density. On the other hand, MRRP, SHR, and TARA, the temperature rise was increased up to 48.6 ${}^{{}^{\circ}}$C at 1000 s and changing nodes, which is impracticable for a human body. Thus by changing network size, the average temperature of proposed protocols performed better.

#### 4.3.6. Average Energy Consumption with Different Node Density

**Figure 10:**
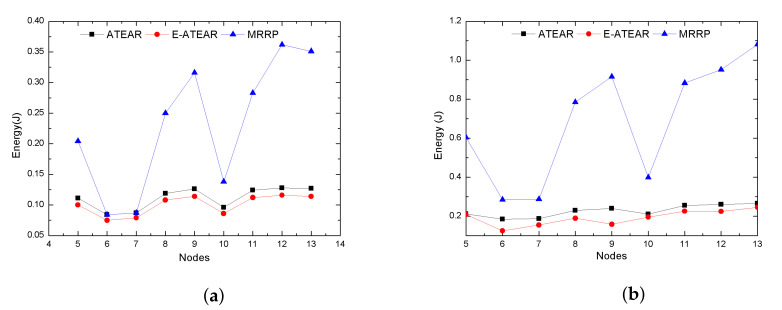
Average energy consumption with different node density. (**a**) Simulation time = 300 s; (**b**) simulation time = 1000 s.

In this work, the change in energy with different simulations and changing the number of nodes was also tested. The results are shown in Figure 10a,b represents the two graphs at two different times 300 s and 1000 s along with the changing in the number of nodes from 5 to 13. The result indicates that energy consumed during 300 s for various simulations and an increasing number of nodes was less (0.075 j to 0.124 j) for two presented protocols while MRRP increased (0.084 j to 0.334 j). Similarly, this behavior was investigated by changing the time from 300 s to 1000 s. The results showed the variation in the average energy of the various simulations by changing the number of nodes at 1000 s. It can be observed that even with more nodes, the maximum average energy consumption for ATEAR and Enhanced ATEAR was 0.71 j and 0.59 j, respectively. The MRRP consumed more energy with increasing nodes, i.e., 1.173 j and at time 1000 s. The proposed protocols performed better than the MRRP.

#### 4.3.7. Throughput with Different Node Density

**Figure 11:**
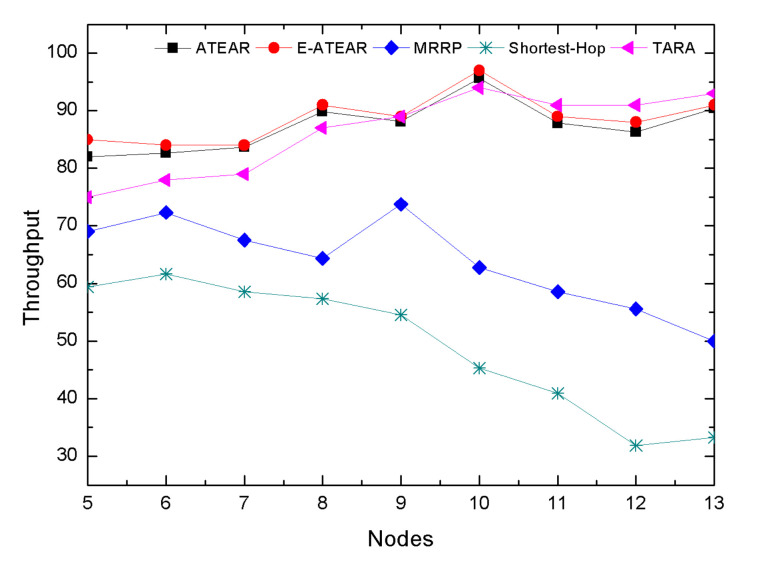
Throughput with different node density.

In Figure 11, we investigated the graphs that represent the throughput comparison of ATEAR, Enhanced-ATEAR, SHR, MRRP, and TARA protocols having a different number of nodes, i.e., 5–13 at simulation time 1000 s. The immediate observation from the graph is that even with the varying network size, ATEAR and Enhanced-ATEAR showed improved performance than MRRP, SHR, and TARA in terms of network throughput. Moreover, it can be analyzed that both ATEAR and Enhanced-ATEAR protocols had a persistent network throughput. In contrast, MRRP, TARA, and SHR the network throughput dropped to 50%, 70%, and 20%, respectively. It can be observed from the graph that the throughput of ATEAR and Enhanced ATEAR was 93% at 1000 s with five nodes. As the throughput is investigated with different networks and node density, it did not drop from the value of 85%, because of the steadiness of temperature and energy consumption the nodes.

### 4.4. WBAN Blockchain System Performance Analysis

**Figure 12:**
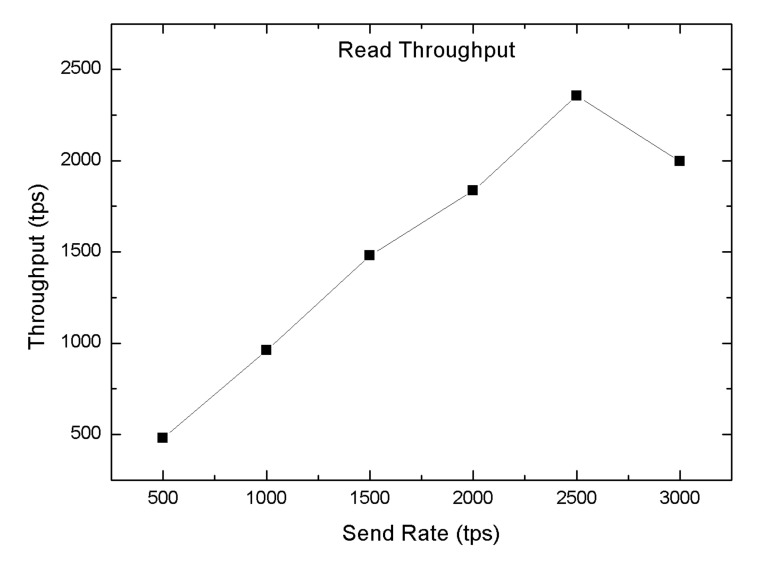
Read transaction throughput.

**Figure 13:**
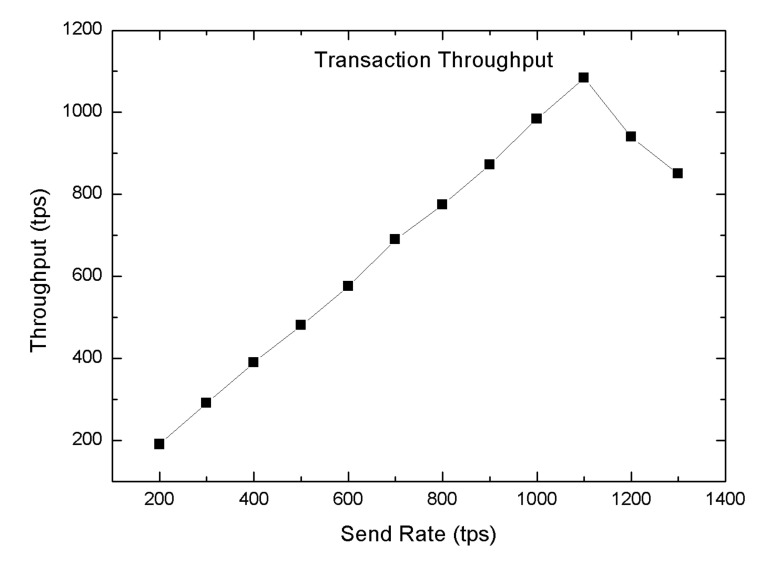
Transaction throughput.

In this section, we carried out several tests to validate the performance of the proposed blockchain-enabled WBAN platform in terms of the block size (number of the transaction), read throughput, transaction throughput, read latency, and transaction latency. We used Hyperledger Caliper, an open-source simulation tool used to investigate the performance of blockchain applications [59]. In order to evaluate the system performance of the blockchain network, we predefined some experimental parameters, i.e., one orderer and four peer nodes. In the proposed blockchain-enabled WBAN platform, we computed the throughput by changing the tps send rate from 200 tps to 1300 tps. The throughput could be broken down into two groups, such as transaction throughput and read throughput. The transaction throughput was defined as the number of transactions triggered in the blockchain network within the assigned time slot, which is also presented in Equation (6). Likewise, the read-through was used to measure the reads operation in the blockchain network within the allocated time slot, as mentioned in Equation (7). The transaction read throughput was measured by varying the tps send rate by 500 to 3000 tps as shown in Figure 12 with random machine utilization configuration. The maximum throughput achieved was 2250 tps with a send rate of 2500 tps. For committing a transaction, the maximum transaction throughput was 1100 tps with a send rate of 1100 tps whereas the lowest one was 205 tps at 200 send rate, also shown in Figure 13.

(6) $$Transaction\;Throughput=\frac{Total\;commited\;transaction}{Time(sec)}$$

(7) $$Read\;Transaction\;Throughput=\frac{Total\;read\;operation}{Time(sec)}$$

**Figure 14:**
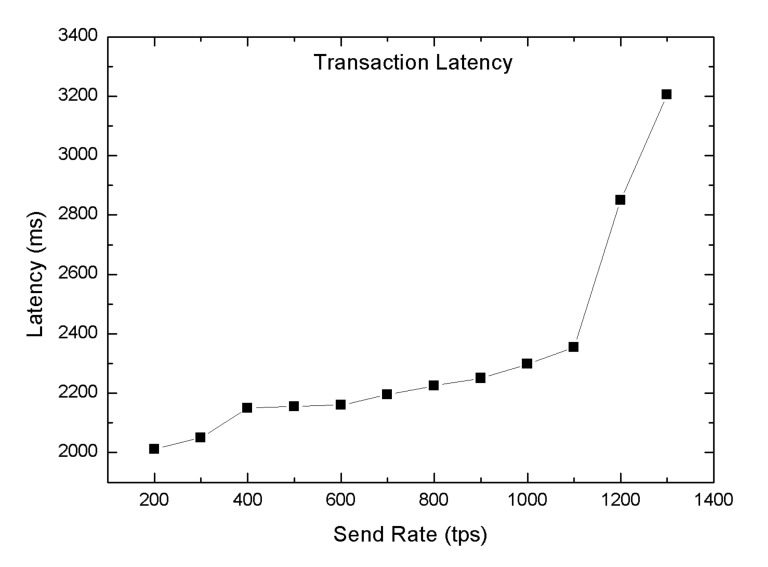
Average transaction latency.

**Figure 15:**
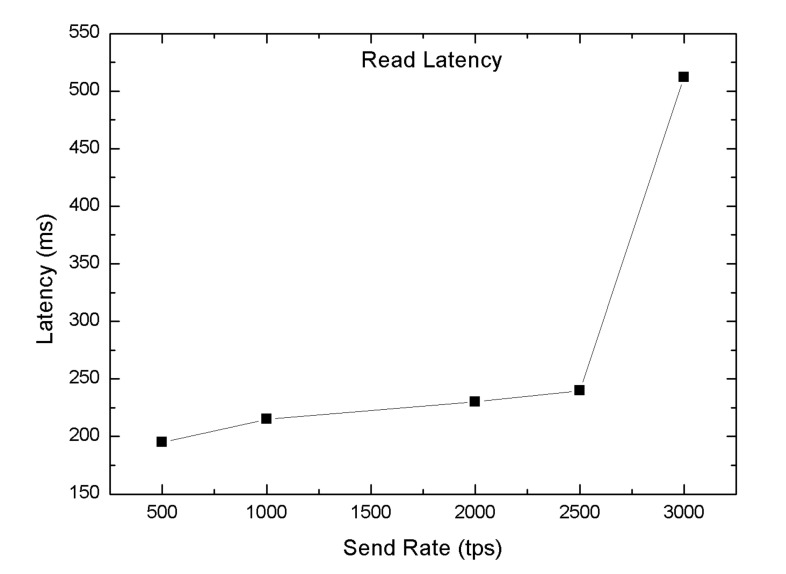
Average read latency.

Figure 14 and Figure 15, show the transaction and read latency for the proposed blockchain-enabled WBAN platform. The term network latency is referred to as the time for a transaction to be executed in the blockchain network. In the proposed system we computed the network latency based on transaction latency and read latency as presented in Equations (8) and (9), respectively. The transaction latency was the amount of time taken by the transaction to be executed across the blockchain network. The time in transaction latency also included transaction submission, broadcast, and transaction consensus mechanism time. Similarly, read latency was measured by considering the round-trip time (RTT) for a transaction to be submitted until its first response. Eyal et al. [60] discuss the network threshold as the network percentile time required to execute the transaction in the network.

(8) TransactionLatency=(Transactionexecutiontime*Networkthresholdtime)−Transactioninvoketime

(9) ReadLatency=(TransactionRTT−transactioninvoketime)

Figure 14 shows the average transaction latency in which the linear rise in the transaction latency occurs as the user request increase in the network. It can be observed from the graph that the transaction latency started rising after 1100 tps send rate, which was considered as the optimal send rate.

Similarly, the average read latency was computed by changing the send rate from 500 to 3000 tps using different machine resources. It can be observed from Figure 15 that the network read latency increased relatively less with the rise of send rate as compared to transaction latency. Nevertheless, the read latency significantly increased after the optimal send rate of 2500 tps.

**Figure 16:**
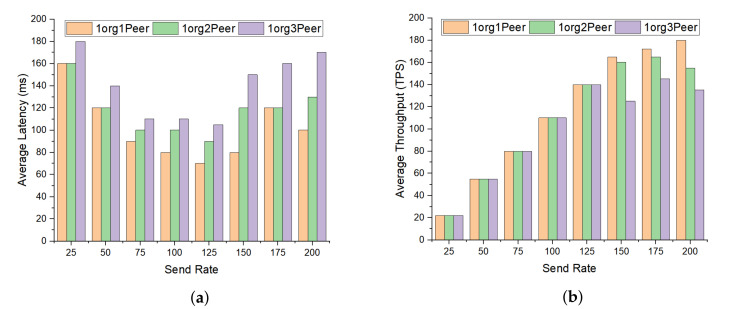
Impact of varying peer node with different transaction rate. (**a**) Average latency; (**b**) average throughput.

Endorser peers endorsed the transaction when it was proposed. The endorser peer function was executed when the specific chaincode was triggered and endorsed the transaction before it was committed. Figure 16, evaluated the impact of varying the number of peer nodes on the performance of the proposed blockchain platform. Figure 16a illustrated the average latency with different peer nodes over the transaction send rate ranges between 25 to 200 tps. It is observed from the graph that the network latency increased with the increase in the number of peers in the network. Moreover, the increase the peer’s nodes also increased the network traffic volume, which ultimately decreased the network throughput as presented in Figure 16b.

**Figure 17:**
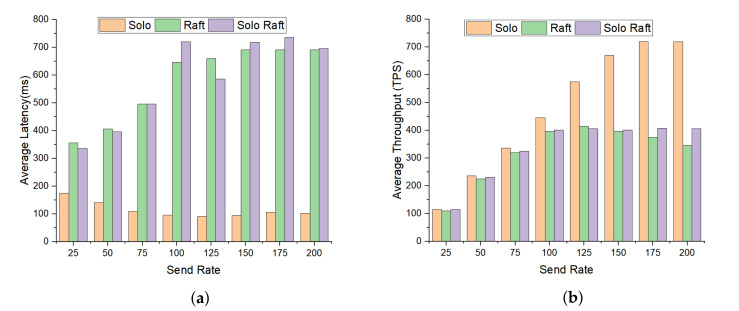
Impact of varying orderer node with different send rate. (**a**) Average latency; (**b**) average throughput.

In Hyperledger Fabric, the ordering of transaction was performed by ordering node along with the other nodes to form ordering service. In Figure 17, we assessed three kinds of ordering service, i.e., solo, raft, and solo raft (the network with single node raft) with varying number of transaction send rate ranges from 25 to 200 transaction per second. It is observed from the graph that the latency of solo ordering was less than the solo raft and raft because solo raft and raft support had additional transport-layer security which provide authentication and security between peer nodes. Figure 17a shows the average latency of the orderer node with a varying number of transaction send rate. Furthermore, the throughput increased linearly as expected until it compressed at 75 tps. The throughput of raft and solo raft was significantly less as compared to solo. The solo ordering showed better application performance than solo raft and raft ordering services since it is a single node and does not require the process of TLS as shown in Figure 16b.

Table 5 shows the resource utilization of the proposed blockchain-enabled WBAN platform in terms of CPU usage, memory utilization, and traffic. For the experimental environment, we used Hyperledger Caliper with five iterations. The peer node in the proposed blockchain network consumed an average memory of 94.32 MB, whereas the average CPU utilization was noted as 5.48%. In the case of the orderer node, the average CPU utilization and average memory consumption was 1.19% and 25.7 MB, respectively. Finally, the certificate authority (ca) node used an average 0% CPU utilization and consumed an average of 5.3 MB memory per processing. Evaluation results illustrate that the proposed protocol performed significantly better in balancing of temperature (to avoid heat damaging effect of the body tissues) and energy consumption (to avoid replacement of battery and to increase the embedded sensor node life) with efficient data transmission achieving a high throughput value. Similarly blockchain-enabled WBAN platform outperformed in terms of resource utilization like memory consumption, machine utilization, and user experience.

## 5. Conclusions and Future Direction

The attaining of good health facility and security of electronic medical records is the most demanding need for mankind, and thus, the WHO emphasized on the fundamental rights of human beings to attain quality healthcare. In recent times, the healthcare system is experiencing transference from a traditional approach to a modernized patient-oriented approach. In a modernized patient-oriented approach, the implementation of the information technology for the exchange of patients’ personal data with ease of communication is in the interest of an individual’s health through telemonitoring and telemedicine systems. Keeping in view all associated advantages, telemonitoring has been recognized as a promising area of research by using the latest tools to transform raw data into useful data. In WBAN, avoiding overheating of the nodes and energy preservation are the two most vital features need to consider for the stability of WBAN. Considering the unique essence of WBAN along with the routing and security issues, a blockchain-enabled routing protocol ATEAR and its improved version Enhanced ATEAR is presented in this work. To testify the significance of the presented protocol, several experiments on the basis of temperature, energy, latency, and throughput are carried out. The results generated from the simulations are compared with the existing routing technique MRRP. The results show a better performance of ATEAR and Enhanced ATEAR than MRRP, TARA, and SHR. The demonstrated results show the temperature rise and energy depletion in MRRP is impracticable for WBAN. Moreover, the proposed blockchain-enabled WBAN platform is based on the network and resolve the existing challenges of WBAN, i.e., scalability, identity, and data security. Temperature and energy, on the other hand, didn’t cause any damage to the tissues due to the fact that the threshold always lies within the threshold. Additionally, the overall performance of the enhanced ATEAR is improved due to energy harvesting and temperature thresholding. The throughput is also considerably improved in the proposed work than in MRRP, TARA, and SHR due to the even distribution of energy and temperature The presented protocols are for fixed implanted sensors with no mobility. Regarding our future work, we are planning to extend the proposed protocol to deal with the different body postures, i.e., the postural movement of the body will be considered along with the maintained temperature and energy of the network.

## Figures and Tables

**Table 1:** State-of-the-art analysis of energy-aware routing approaches.

Methods	Objective	Limitations
Co-CEStat [20]	Energy efficient routing protocol withhigh network throughput and lifetime. Support dynamic routing.	Energy utilization is high.
MEPF [21]	Minimize energy of node with less transamission power.	Network latency is high.
RSSI [22]	High energy consumption with QoS service	Packet loss is high.
DARE [32]	Minimize energy consumption of node and diminish the node hotspot.	Load distributed in not uniform.
ESR [33]	Improve patient mobility and traffic load.	Network life cycle is less.
SIMPLE [34]	Energy consumption is balanced among all nodes. High network throughput.	Packet drop is high.

**Table 2:** State-of-the-art analysis of temperature-aware routing protocols.

Methods	Objective	Limitations	Domain
TARA [45]	Compute the temperature-rise of nodes and redirect the packet from hotspot area.	Failed to provide hotspot avoidance. High node temperature with less network life cycle.	Cancer and retinal detection
LTRT [43]	Temperature-rise is less. Packet rate is high and throughput.	Network life cycle is less with no hotspot avoidance.	Monitoring system for cardiac patients
RAIN [42]	Efficiently route selection toward sink in an id-less BSN. Diminish the problem of hotspot.	Delay in packet delivery.	In-vivo network of implanted sensors nodes
M-ATTEMPT [38]	To route the packet away from hotspot area.	Failed to select new route in case of hotspot. Load distribution on node is not uniform.	Homogeneous and Heterogeneous WBAN
Re-ATTEMPT [38]	To route the packet away from hotspot area and increase network life.	The network life cycle is less	Homogeneous and Heterogeneous WBAN
HPR [40]	Diminish the problem of hotspot in network.	The network life cycle is less	Heterogeneous WSN’s
THSR [41]	To minimize the temperature of the node and avoid hotspot creation.	Network life cycle is less	Heterogeneous WSN’s
LTR [44]	To diminish the temperature of the node in a network and route the packet away from the hotspot	Packet throughput is less. High node temperature. Network life time is less	Monitoring system for cardiac patients
M2E2 [37]	To route the packet away from hotspot area and increase the network life in wireless body sensor networks.	High node temperature.	Heterogeneous WSN’s
ALTR [44]	To minimize the temperature of the node in a network	High end-to-end delay with less network life. Packet drop throughput is less.	Monitoring system for cardiac patients

**Table 3:** Ranking of nodes.

Node Temperature	Node Energy	Assigned Rank to Temperature ($R_{\mathrm{TN}}$)	Assigned Rank to Energy ($R_{\mathrm{EN}}$)	Assigned Rank to Nodes ($R_{\mathrm{AN}}=R_{\mathrm{TN}}+R_{\mathrm{EN}}$)
37 ${}^{{}^{\circ}}$C	0.5 j	1	1	2
38.2 ${}^{{}^{\circ}}$C	0.38 j	4	6	10
38 ${}^{{}^{\circ}}$C	0.44 j	3	4	7
39 ${}^{{}^{\circ}}$C	0.47 j	9	2	11
38.5 ${}^{{}^{\circ}}$C	0.45 j	5	3	8
38.7 ${}^{{}^{\circ}}$C	0.4 j	6	5	11
38.9 ${}^{{}^{\circ}}$C	0.35 j	7	7	14
37.9 ${}^{{}^{\circ}}$C	0.33 j	2	8	10

**Table 4:** Simulation setup.

Component	Description
Simulation Area	3 cm × 2 cm
Implanted nodes count	9
Sink Node	1 static
Node Initial energy	0.3 j
Initial temperature of a node	37 ${}^{{}^{\circ}}$C
Size of a Packet	296 bits
Threshold temperature value	40 ${}^{{}^{\circ}}$C
Threshold energy value	0.1 j
Application type	Event-Driven

**Table 5:** Resource use analysis of proposed system.

Type	Name	CPU	CPU	Memory	Memory	Traffic	Traffic
		(max %)	(avg %)	(max)	(avg)	In	Out
Docker	peer0.com	12.44%	5.59%	106.6 MB	98.5 MB	4 MB	4.2 MB
Docker	peer1.com	17.09%	6.24%	93.5 MB	85.7 MB	4.3 MB	5.2 MB
Docker	peer2.com	15.02%	4.56%	110.5 MB	105.3 MB	5.6 MB	10 MB
Docker	peer3.com	0.00%	5.54%	90.8 MB	85.8 MB	4.8 MB	5 MB
Docker	orderer.com	4.95 %	1.15%	34.5 MB	25.7 MB	5 MB	10.6 MB
Docker	ca_0	0.00%	0.00%	5.5 MB	5.2 MB	546 B	0 B
Docker	ca_1	0.00%	0.00%	5.2 MB	5.2 MB	430 B	0 B
